# Reconstructing the metastatic journey: functional circulating tumor cells and disseminated tumor cells based models for translational oncology

**DOI:** 10.1186/s13046-025-03617-y

**Published:** 2026-01-14

**Authors:** Caroline Sanglier, Laure Cayrefourcq, Catherine Alix-Panabières

**Affiliations:** 1 Laboratory of Rare Human Circulating Cells (LCCRH), University Medical Centre of Montpellier, Site Unique de Biologie (SUB), 371, Avenue du Doyen Gaston Giraud, 34295 Cedex 5 Montpellier, France; 2 CREEC/CANECEV, MIVEGEC (CREES), CNRS, IRD, University of Montpellier, Montpellier, France; 3 European Liquid Biopsy Society (ELBS), Hamburg, Germany

**Keywords:** CTC, CTC lines, PDX models, Liquid biopsy, DTC

## Abstract

Metastatic progression, driven by the dissemination of circulating tumor cells (CTCs) through the bloodstream, remains the leading cause of cancer-related death. A rare subset of CTCs, characterized by tumor-initiating properties and phenotypic plasticity, plays a pivotal role in the formation of distant metastases. The ability of these cells to survive in the circulation, evade the immune surveillance, and establish secondary tumors underscores their biological significance. However, CTC extreme rarity and heterogeneity pose major challenges for their in-depth functional characterization. Disseminated tumor cells (DTCs) are cells that have extravasated and persist in distant organ niches, often in a dormant state, and represent a complementary and equally critical component of metastatic progression. Their capacity to remain quiescent for prolonged periods before reactivation highlights the need to study both CTCs and DTCs to fully understand metastasis initiation and relapse. Recent advances in CTC isolation and culture have led to the development of patient-derived CTC lines and CTC-derived xenograft animal models, offering unprecedented opportunities to investigate metastatic seeding, therapeutic resistance and tumor evolution. CTC- and DTC-based models provide valuable insights into the biology of CTCs from different cancer types, revealing key molecular drivers of metastasis formation and potential therapeutic targets. In this review, we summarize the state-of-the-art methodologies for establishing CTC- and DTC-based models and evaluate their contribution to understand tumor progression and response to treatments. We discuss the current challenges in generating and maintaining these models, including the influence of hypoxic conditions, enrichment strategies, and culture medium optimization. Then, we highlight their potential applications in precision oncology, particularly for biomarker discovery and for preclinical drug testing.

## Background

Metastasis formation is the process by which tumor cells spread from their primary site to distant organs, primarily through the bloodstream. This process is responsible for the vast majority of cancer-related deaths [1]. Despite decades of research, the biology of tumor dissemination remains only partially understood, and effective strategies for prevention and treatment are still limited. Circulating tumor cells (CTCs) are among the key players in this process. CTCs actively detach from the primary tumor and travel through the circulation. CTCs were first observed more than 150 years ago, but only recently their critical role in metastasis has been highlighted [2, 3]. These cells are extremely rare in the blood, and only a small fraction possesses the ability to survive the harsh conditions in the bloodstream, infiltrate distant organs (seeding), and ultimately form metastases. This highly specialized subset, capable of initiating metastatic growth, is often referred to as metastasis-initiating cells [4].

Besides CTCs, disseminated tumor cells (DTCs) are increasingly studies. DTCs have successfully extravasated and lodged in distant tissues. Unlike CTCs, DTCs can persist in a dormant state for months or years before reactivation to form overt metastases [5]. Their biology is closely intertwined with that of CTCs, and studying both cell types offers essential complementary insights into the metastatic cascade. Understanding how CTCs survive in circulation and how DTCs adapt to foreign microenvironments is crucial to unraveling early metastatic events and therapeutic resistance.

In recent years, technological advances have transformed our ability to detect and analyze CTCs at the molecular level, opening new possibilities for personalized therapeutic strategies [6, 7]. Moreover, several tools have been developed to establish ex vivo CTC cultures, each with its advantages and disadvantages. Establishing such cultures remains challenging and time-consuming, but the resulting in vitro models provide a unique and controlled environment to investigate the biology of metastasis-competent CTCs and their response to therapeutic agents. These systems offer critical insights into the cellular mechanisms of metastatic spread and treatment resistance [8–11]. For example, testing drug responses in these models can help to identify the most promising compounds for clinical development and even guide personalized therapy decision-making [12, 13].

In this review, we explore the different experimental models developed to study CTCs and DTCs, including long-term cell lines and CTC-derived xenografts (CDXs). For each cancer type, we examine how these models were established, their contribution to our understanding of cancer cell dissemination, and their role in the design of innovative treatments. We particularly focused on their contribution to unravel the mechanisms driving metastasis and to better understand CTC and DTC behavior in different biological and therapeutic contexts.

### Establishment of CTC lines from solid cancers

**Fig. 1:**
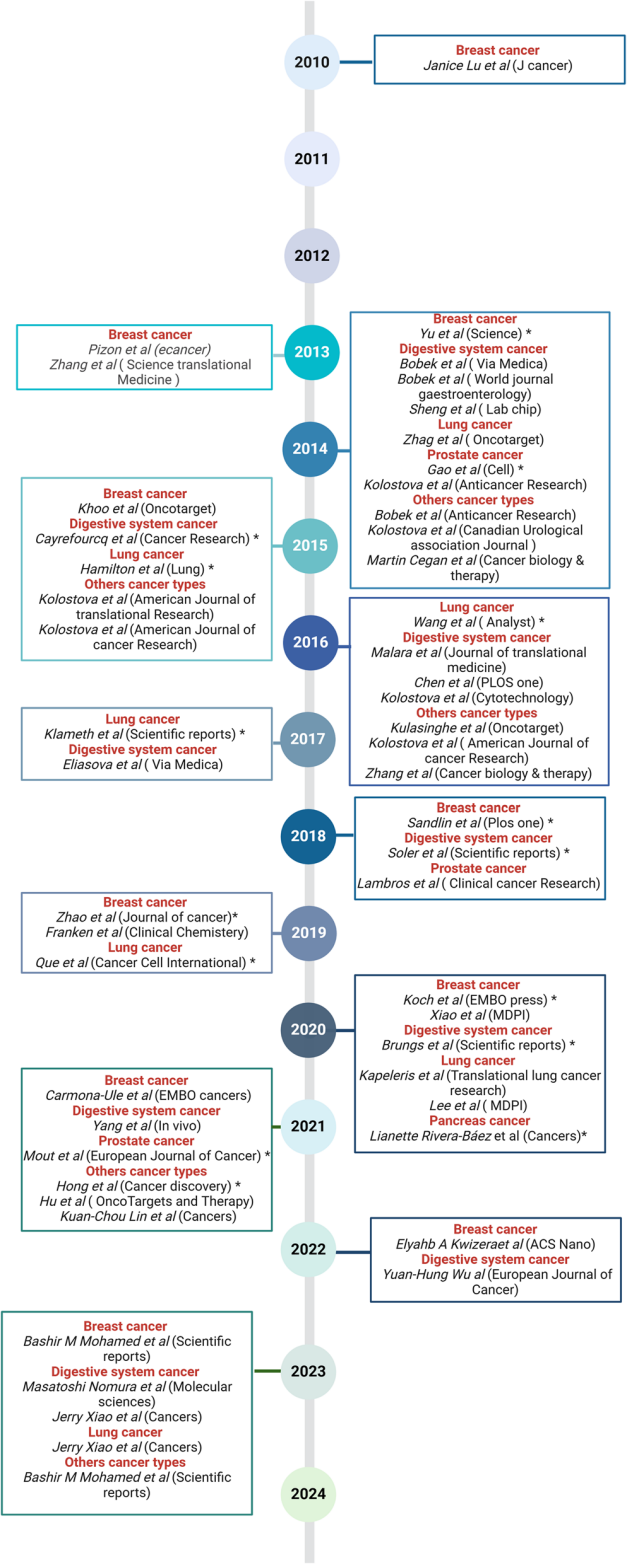
Timeline of circulating tumor cell (CTC)-derived cell line establishment (short-term and long-term cultures). Each point represents the first publication reporting the derivation of a CTC line. Asterisks (*) indicate long-term CTC lines; entries without asterisk correspond to short-term cultures. The cancer type is specified next to each point, as well as the first author and the journal name

In this first chapter, we describe several studies on the in vitro culture of CTCs, focusing mainly on the methods used to establish these cell lines and the culture conditions. We grouped studies according to the tumor type, because of the specific features of each cancer type. We considered a cell culture to be long-term when the culture period exceeded 6 months. The data extracted from these studies are summarized in Tables 1 and 2. Figure 1 provides an overview of the chronological progression of published studies, illustrating the timeline of long-term and short-term CTC culture establishment in different cancer types.

**Table 1:** List of the available permanent or long-term CTC lines

Cancer type		CTC line name	Culture duration	Year	Success rate	CTC enrichment and isolation	Blood volume	Culture conditions	Ref.
Breast cancer		BRx33, BRx07, BRx68, BRx50, BRx42, BRx61	6–12 months	2014	6/36	CTC-iChip	6 to 18 mL	4% O_2_	[14]
		BRx82, BRx142	> 6 months	2018	/		/	4% O_2_	[15]
		CTC-3	2 years	2019	1/16	RosetteSep™	6 mL	Normoxic conditions, 5% CO_2_	[16]
		CTC-ITB-01	4 years	2020	/		7.5 mL	Normoxic conditions, 5% CO_2_	[17]
Digestive cancer types	Colon cancer	CTC-MCC-41	> 6 months	2015	1/50	RosetteSep™	10 mL	Initial environment: 2% O_2_. Maintenance: 5% CO_2_, normoxic conditions	[18]
		CTC-MCC-41.4, 41.5 A. 41.5B. 41.5 C, 41.5D. 41.5E, 41.5 F, 41.5G	> 6 months	2018	/		10 mL	Normoxic conditions	[19]
	Gastroesophageal cancer	UWG01CTC, UWG02CTC	> 12 months	2020	2/23		15 mL	Hypoxic conditions	[20]
	Pancreatic cancer	/	> 6 months	2020	3/NC	Labyrinth microfluidic device	/	3D hanging drop array plates and 2D culture in 5% CO_2_	[21]
Lung cancer	Lung adenocarcinoma	N/A (one line was named CTC-TJH-01 in the article [22])	> 6 months	2017	2/35	Microfluidics-based immunomagnetic isolation. EpCAM- and EGFR-coated immunomagnetic microbeads	2 mL	3% O_2_, 5% CO_2_ (1–14 days), then 5% CO_2_, normoxic conditions	[23]
	Small cell lung cancer	BHGc7, BHGc10, BHGc26, BHGc27, BHGc50, BHGc59, BHGc71, UHGc5	> 4 months; long-term culture was confirmed by two follow-up studies	2015–2016	/	Ficoll-Hypaque density gradient	/	Normoxic conditions	[24, 25]
	Non-small cell lung cancer	CTC-TJH-01	24 months	2019	2/89	A mixture of EpCAM- and EGFR-coated immunomagnetic microbeads in Herringbone Microfluidic Chip	5 mL	Normoxic conditions, 5% CO_2_	[22]
Prostate cancer		MSK-Pca1–7	Weekly passaging at 1:3 ratio	2014	1/17	RosetteSep™ Human CD45 Depletion Cocktail	8 mL	/	[26]
		EMC-Pca-41	> 1 year	2021	1/40	Leukapheresis, RosetteSep™ Human CD4 Depletion Cocktail	< 10 L	Normoxic conditions	[27]
Melanoma		Mel-167, PEM-22, Mel-182, PEM-78	Long-term	2021	4/37	CTC-iChip	10 mL	Hypoxia, 5% CO_2_, 4% O_2_	[28]

**Table 2:** List of the available short-term CTC lines

Cancer type		Culture duration	Year	Success rate	CTC enrichment and isolation	Blood volume	Culture conditions	Ref
Breast cancer		3 days	2010	21/29	Ficoll-Paque density gradient	10 mL	37 °C, 5% CO_2_	[29]
		Several passages	2013	3/38	FACS (CD45, ALDH1, EpCAM)	20 to 45 mL	37 °C, 5% CO_2_	[30]
		28 days	2013	31/39	Red blood cell lysis	1 mL	37 °C, 5% CO_2_	[31]
		2–8 weeks	2015	/	Red blood cell lysis	10 mL	37 °C, 5% CO_2_, 1% O_2_. normoxic conditions	[32]
		> 3 months	2019	2/8 (no cryopreserved samples), 3/9 (cryopreserved samples)	Diagnostic leukapheresis, Parsortix^®^ microfluidic system	3.41 L	5% CO_2_, 4% O_2_	[33]
		> 30 days (6 samples), < 30 days (6 samples)	2020	12/12	Ficoll-Paque density gradient	7.5 mL	37 °C	[34]
		> 23 days (up to 291 days, mean = 8 weeks)	2021	36/80	RosetteSep™	7.5 mL	37 °C, 5% CO_2_, 1–2% O_2_ (1 week), then, normoxic conditions	[35]
		35–60 days	2022	36/60	VIZA microfluidic chip	1 mL	37 °C, 5% CO_2_	[36]
		> 8 weeks	2023	/	ScreenCell^®^ Kit LCD	6 mL	/	[37]
Digestive cancer types	Hepatocellular carcinoma	> 7 days	2016	31/36 (> 100 μm defined as spheroids)	Microfluidic chip, cell capture and release	2 mL	37 °C	[38]
		12–14 days	2021	55 (spheroids)/60 (CTCs)/106 (total)	Ficoll-Paque density gradient	5 mL	37 °C, 5% CO_2_	[39]
	Colon cancer	14 days	2016	/		5 mL	/	[40]
	Colorectal cancer	10 days to 2 months	2016	/	Anti-EpCAM antibody conjugated lipid-coated microfluidic device (CMx platform), gentle air foam stream	2 mL	/	[41]
		3–5 days, some up to 6 months	2017	81/98	Size-based MetaCell^®^: filtration using a porous polycarbonate membrane (pores of 8 μm in diameter)	8 mL	37 °C, 5% CO_2_	[42]
		7 and 14 days	2023	18/33	OncoQuick^®^ density gradient + adhesion capture on PMEA-coated plates	10 mL	/	[43]
	Esophageal cancer	< 14 days	2014	27/43	MetaCell^®^: filtration through a porous polycarbonate membrane (pores of 8 μm in diameter)	8 mL	37 °C, 5% CO_2_	[44]
	Gastric cancer	> 14 days	2016	13/22		8 mL	37 °C, 5% CO_2_	[45]
	Pancreatic cancer	> 14 days	2014	16/24		8 mL	37 °C, 5% CO_2_	[46]
		NA	2014	0/12	GEM Chip	5–10 mL	37 °C, 5% CO_2_	[47]
		3 weeks	2022	/	RosetteSep™	20-mL	/	[48]
		14–28 days	2023	23/25	Ficoll-Paque density gradient	7–8 mL	37 °C	[49]
	Gastrointestinal cancer	4 weeks	2021	13 (colonies)/38 (viable cells)/81 (total)	Red blood cell lysis	5 mL	37 °C, 5% CO_2_	[50]
Lung cancer	Lung cancer	NA	2014	14/19	Microfluidic device for CTC capture	5 mL	37 °C, 7.5% CO_2_	[51]
	Non-small cell lung cancer	20 to 50 days	2020	9/70	RosetteSep™	10 mL	1–2% O_2_	[52]
	Small cell lung cancer	2 to 6 weeks	2020	18/22	RosetteSep™	7.5 mL	/	[53]
	Lung cancer	> 14 days	2023	/	Ficoll-Paque density gradient	6 mL	37 °C	[54]
Prostate cancer		1 week to 3 months	2009	5/8	Ficoll-Paque	3 mL	/	[55]
		14 days	2014	18 (proliferative capacity)/28 (CTC-positive)/55 (total samples)	MetaCell^®^: filtration through a porous polycarbonate membrane (pores with a diameter of 8 μm)	8 mL	37 °C, 5% CO_2_	[56]
		> 4–6 weeks	2018	2/14	Apheresis, EasySep™ Human EpCAM Positive Selection Kit	40 to 100 mL	/	[57]
Other cancer types	Gynecological cancer	3–10 days	2015	3/3	/	8 mL	37 °C, 5% CO_2_	[58]
	Ovarian cancer	3–14 days	2015	77/118	/	8 mL	37 °C, 5% CO_2_	[59]
	Pleural mesothelioma	10–14 days	2014	45/50	MetaCell^®^: filtration through a porous polycarbonate membrane (pores of 8 μm in diameter)	8 mL	37 °C, 5% CO_2_	[60]
	Urothelial tumors	10–14 days	2014	/		8 mL	37 °C, 5% CO_2_	[61]
	Bladder cancer	40 days	2014	9/70	MetaCell^®^: filtration through a porous polycarbonate membrane (pores of 8 μm in diameter)	15–20 mL	Hypoxic	[62]
	Head and neck cancer	4 weeks	2021	/	Ficoll-Paque and RosetteSep™	10 mL	/	[63]
	Head and neck cancer	14 days	2016	7/25	RosetteSep™	10 mL	/	[64]
	Ovarian cancer	103 days	2015	/	MetaCell^®^	5 mL	/	[58]
	Ovarian cancer	8 weeks	2023	/	Red blood cell lysis	/	37 °C, 5% CO_2_	[37]

### Methodological note on sampling time

To designate an in vitro culture as a genuine CTC line, blood collection should be performed before any invasive tumor procedures (e.g., surgery, biopsy and other invasive procedures). It is well established that tumor handling and the perioperative period induce a transient surge of tumor cells in the circulation, often as single cells or clusters [65, 66]. As these cells are mechanically dislodged by the intervention, strictly speaking, they cannot be classified as “CTCs.” Unlike *bona fide* CTCs that have actively entered the bloodstream and may possess metastatic potential, these iatrogenically released cells are simply shed from the primary tumor. Consequently, blood samples collected after invasive procedures contain predominantly these artifactually released cells rather than true CTCs or circulating metastasis-initiating cells. Therefore, when establishing CTC-derived models, it is essential to report precisely the timing of blood collection relative to anesthesia, incision, and other surgery steps [67–71].

#### Breast cancer

Between 2014 and 2020, only four research teams managed to establish long-term breast cancer CTC lines, using two main enrichment strategies: (1) the CTC-iChip platform and (2) the RosetteSep™ CD45 depletion method.

The CTC-iChip is a microfluidic device that combines deterministic lateral displacement, inertial focusing, and magnetophoresis. It can sort up to 10^7^ cells per second and displays a capture efficiency of ~ 97% for rare cells [72]. This high-throughput technology, first used by Min Yu et al.. (2014), can isolate CTCs from small blood volumes (~ 7–18 mL) and allowed establishing several breast cancer CTC lines (e.g., BRx33, BRx07, BRx68, BRx50, BRx42, BRx61) [14]. These CTC lines kept the molecular traits of the original tumors (e.g., ER^+^ status and PIK3CA, TP53, KRAS, FGFR2 mutations) after long-term culture (> 6 months). They have been crucial to study breast cancer plasticity and drug resistance and served to build preclinical models of metastatic progression. Moreover, Yu et al. showed that some of these CTC lines could be transplanted into mice (xenografts). Specifically, 3/5 BRx CTC lines formed tumors in mice, confirming their tumorigenic potential and providing a system to study breast cancer metastasis formation in vivo [14]. A longitudinal genomic analysis revealed accumulation of mutations upon exposure to chemotherapy drugs [14], suggesting that tracking mutations in cultured CTCs could guide adaptive personalized therapy. Cultured breast cancer CTCs often have a mixed epithelial/mesenchymal phenotype and express stem cell markers (e.g., CD44, CD24, ALDH1), reflecting the tumor plasticity and aggressive subpopulations. Importantly, Sandlin et al. (2018) reported that the BRx-142 CTC line could be cryopreserved without losing viability or traits, enabling long-term functional studies [15].

The RosetteSep™ enrichment method is based on the negative selection of normal blood cells or depletion of leukocytes. An antibody cocktail causes leukocytes (e.g., CD45⁺, CD36⁺ cells) to form immune rosettes with red blood cells (RBC) that are then removed by density gradient centrifugation. Thus, intact CTCs, not bound to rosettes, are enriched for culture. This method allowed establishing two metastatic breast cancer CTC lines between 2019 and 2020: CTC-3 and CTC-ITB-01. The CTC-3 line was derived from the blood sample of one of the fifty included patients (success rate: ~2%) and was cultured for > 2 years [16]. It strongly expresses CD44, suggesting a tumor stem cell phenotype, and can produce xenografts in mice. The CTC-ITB-01 line (Koch et al., 2020) was maintained in culture for > 4 years [17]. Interestingly, CTC-ITB-01 cells expressed epithelial markers (E-cadherin, EpCAM, K19 and CD24) and also epithelial-mesenchymal transition (EMT) markers (Twist1), showing a hybrid state likely linked to CTC dissemination ability. Koch et al. found that CTC-ITB-01 cells could grow as adherent monolayers and non-adherent spheroids, switching between conditions, like cancer stem cells with adhesion/de-adhesion plasticity [17]. The RosetteSep™ method, although less efficient (some leukocytes remain), proved effective for viable CTC recovery and long-term CTC culture. These models were used for molecular profiling and drug sensitivity testing to study resistance mechanisms and find new treatment targets.

All long-term breast cancer CTC lines were initially grown in standard culture conditions (normoxia: ~21% O₂, 5% CO₂, 37 °C). Most protocols use growth factor-enriched media (e.g., EGF, bFGF, insulin/transferrin, B27/N2 supplements) and 3D-friendly surfaces (ultra-low attachment plates, Matrigel) to encourage spheroid/organoid formation rather than 2D monolayers. Yu et al. found that CTCs often senesce after few divisions in 2D adherent culture, while continuous proliferation is promoted in 3D suspension [14]. Thus, spheroid growth (often in serum-free medium with EGF/FGF and the anti-apoptotic Y-27632 ROCK inhibitor) has become the standard approach to initiate breast cancer CTC cultures.

Some studies tested hypoxia effects on CTC culture success. For example, Carmona-Ule et al. (2021) cultured RosetteSep™ CD56-enriched breast cancer CTCs in 1–2% O₂ for 1 week to favor “stem-like” cell survival, and then switched to normoxia for expansion [35]. Hypoxia may protect dormant/quiescent tumor cells and promote tumor stem cell enrichment. However, hypoxia does not seem to be strictly required. Indeed, a 2024 review found that ~ 2/3 of studies started CTC culture in hypoxic conditions (2–8% O₂) and ~ 1/3 in normoxic conditions with similar results [73]. Thus, while hypoxia can enhance survival of dormant or stem-like CTCs, its non-essential role suggests that the intrinsic tumor cell features and other culture conditions might be the critical determinants of successful CTC expansion. Hypoxia can help to reduce oxidative stress and favor the survival of fragile CTCs at the beginning; however, long-term success depends on the intrinsic biology of the patient’s CTCs. Many “hypoxia” protocols gradually return to 20% O₂ once CTC clusters start proliferating. Short-term ex vivo cultures (< 1 month) often are maintained in standard conditions (ambient air, 5% CO₂) and colonies/spheroids are observed after 1–3 weeks. Almost all studies reported the successful establishment of temporary CTC cultures from a significant fraction of blood samples, but stable long-term lines (> 6 months) only in < 10% of cases. Short-term cultures are still useful for quick phenotypic tests (e.g., drug sensitivity, tumorsphere formation as an aggressiveness marker). Overall, optimizing 3D vs. 2D cultures, normoxia vs. hypoxia, and enriched media will improve the chances, but success mainly depends on the patient-specific CTC biology.

The initial blood volume to isolate breast cancer CTCs varies widely and affects the yield of rare CTCs. Many of the successful CTC lines came from moderate blood volumes, typically 6–18 mL. A standard ~ 7.5 mL tube is often used with the density gradient (Ficoll-Paque™) or RosetteSep™ methods. For instance, Janice Lu et al. (2010) [29], Xiao et al. (2020) [34], and Carmona-Ule et al. (2021) [35] enriched CTCs from a single 7.5 mL blood sample, showing that small blood amounts can work with optimized protocols. Other authors used much larger blood volumes to maximize CTC yield. Zhang et al. (2013) used fluorescence-activated cell sorting (FACS) (EpCAM⁺/ALDH1⁺/CD45⁻ cells) and 20–45 mL blood per patient to target rare ALDH1⁺ stem-like CTCs linked to dormancy [30]. Franken et al. (2019) combined diagnostic leukapheresis, in which the patient blood is filtered for ~ 2 h, with Parsortix™ microfluidics, processing up to 3.41 L per patient to concentrate CTCs [33]. This enabled the establishment of 3D CTC cultures from patients where standard methods failed, using 4% O₂ and non-adherent “tumorsphere” media. The disadvantage of this method is the heavy procedure, unsuitable for routine applications, but valuable in research.

Between these extremes (7.5 mL vs. L), there are also mid-range protocols (~ 50–100 mL). Some simplified methods avoid enrichment steps, for instance RBC lysis solutions to remove erythrocytes and keep nucleated cells (CTCs + leukocytes). Pizon et al. [31] and Khoo et al. [32] showed that in good conditions, RBC lysis preserves CTC viability and allows CTC colony/spheroid formation from 5 to 10 mL blood samples without the need of density gradient. The tradeoff is lower CTC purity (more white blood cells), which can interfere with CTC expansion unless selective conditions are used (e.g., 3D culture, low serum). In summary, sensitivity and practicality must be balanced when choosing the blood volume and isolation method. Large blood volumes and CTC selection methods increase the success rate, but need more resources. Single-tube standard methods are simpler, but have lower success rates, especially when using blood from patients with low CTC count.

Recently, new technologies have been developed to boost CTC culture success by exploiting their biological heterogeneity. One major innovation is the Virtually Implemented Zones of Attraction (VIZA) microfluidic chip developed by Elyahb A. Kwizera et al. (2022) [36]. In this chip, the PDMS surface is coated with a gold nanolayer and thiol-gold chemistry is used to attach capture antibodies against both EpCAM and CD44. The results are striking: capturing EpCAM^+^ and CD44^+^ CTCs increased the percentage of blood samples from patients with stage IV breast cancer that led to culturable/multipliable CTCs from ~ 3–16% (standard EpCAM capture) to nearly 70%. Historically, > 80% of CTCs from patients with metastatic breast cancer fail to grow even after isolation; the VIZA approach overcomes this hurdle in most cases.

Other new devices can be used for CTC isolation. In 2023, the ScreenCell^®^ system gained popularity as a simple, cheap, label-free microfiltration method [37]. ScreenCell^®^ devices use calibrated microporous membranes to retain tumor cells based on their size/flexibility compared with normal blood cells. Despite some stress, ScreenCell^®^-isolated CTCs are viable for short-term culture and high-quality DNA/RNA extraction. Its appeal lies in preserving tumor cell morphology and in being antigen-independent, thus avoiding the loss of EpCAM-negative or phenotype-shifted CTCs.

#### Digestive cancer

A 2015 study described the first successful long-term CTC line (CTC-MCC-41) derived from the blood of a 57-year-old patient collected at the diagnosis of metastatic colon cancer [18]. This pioneer colon CTC line exhibited epithelial and stem-like traits and mirrored key genomic characteristics of the patient’s primary tumor and lymph node metastasis. The same research group obtained eight additional CTC lines from the same patient at subsequent time points during treatment and disease progression. These included one CTC line after therapy (CTC-MCC-41.4) and seven CTC lines from a blood sample collected before the patient’s death (CTC-MCC-41.5 A to 41.5G). This unique longitudinal panel (nine lines in total) enabled the study of clonal selection and therapy-induced adaptation over time in a real patient. Comparative analyses showed that in the CTC lines established after therapy, genes related to drug resistance and metabolism were upregulated, reflecting how treatment pressure shapes the tumor cell phenotype [19]. All these colon CTC lines were cultured for long time (many months) and could be continuously propagated, providing a valuable model of metastatic colorectal cancer evolution and resistance mechanisms. In 2020, another group established two long-term CTC lines (UWG01CTC and UWG02CTC) from two patients with metastatic gastroesophageal neuroendocrine carcinoma and metastatic gastroesophageal adenocarcinoma, respectively [20]. These CTC lines had distinct phenotypes reflecting the patient’s tumor biology: one CTC line (UWG02CTC) was EpCAM^+^, cytokeratin^+^ and CD44⁺, consistent with an epithelial tumor profile. The other line (UWG01CTC) came from a patient with high-grade neuroendocrine carcinoma and was EpCAM^−^ with weak cytokeratin expression, but strong neuroendocrine marker expression. Upon injection into mice, both CTC lines displayed rapid xenograft growth, confirming their viability and malignancy. Moreover, they showed differential sensitivities to drugs and radiation therapy that matched the differences in the patient’s tumors. These CTC lines were cultured in hypoxic conditions in order to mimic the bloodstream environment and promote survival of oxygen-sensitive CTCs. The use of a negative-selection enrichment (RosetteSep™) method was crucial to capture both EpCAM^−^ and EpCAM^+^ CTCs, a strategy that avoids reliance on epithelial markers and thereby includes more mesenchymal and neuroendocrine tumor cells. The UWG01CTC and UWG02CTC lines demonstrate that even CTCs with non-epithelial phenotypes can be grown for a long time by using appropriate isolation techniques and culture conditions.

Long-term CTC cultures have been established also from blood samples of patients with pancreatic cancer using an innovative size-based microfluidic isolation. In 2020, Rivera-Báez et al. employed the Labyrinth microfluidic device, a label-free inertial flow system, to enrich CTCs from blood samples of ten patients with advanced pancreatic ductal adenocarcinoma [21]. They could expand CTCs from three patients (as adherent cells and spheroids) and used these cultured CTCs to generate CTC-derived xenograft (CDX) models in mice. The cultured pancreatic CTC lines harbored KRAS mutations typical of pancreatic tumors, confirming their origin. Upon injection in mice, the tumor take rate was 100%, demonstrating robust tumorigenic potential. This provided proof that pancreatic cancer CTCs, once expanded ex vivo, can form tumors in vivo and may serve as a platform for testing targeted therapies in an individualized manner. All CTC lines, from colon, gastroesophageal and pancreatic cancer, were established using enrichment by negative selection (e.g. RosetteSep™) or label-free size-based capture to avoid selection based on EpCAM expression. Such methods maximize the recovery of viable CTCs with different phenotypes.

Even when permanent cell lines cannot be derived, short-term CTC cultures (from days to weeks) have been obtained using various techniques. These transient CTC cultures are extremely valuable for assessing CTC morphology, viability, and drug responses before senescence. Researchers have explored a range of enrichment and culture methods to maintain CTCs ex vivo from patients with gastrointestinal malignancies: density-gradient enrichment, EpCAM-targeted microfluidic culture, 3D spheroid cultures of CTCs, size-based filtration and extended culture, hepatocellular carcinoma (HCC)-specific microfluidic chip, RBC lysis and feeder co-culture, and Geometrically Enhanced Mixing (GEM) chip for pancreatic CTCs. Briefly, density-gradient enrichment (Ficoll-Paque™ and OncoQuick™) is a simple centrifugation-based method that can enrich viable CTCs by separating mononuclear cells from whole blood. Using Ficoll-Paque™, colon cancer CTCs have been maintained in vitro for up to ~ 14 days in short-term culture. For example, one study reported that using Ficoll isolation, all tested blood samples with CTCs from patients with colon cancer yielded short-lived CTC cultures (up to 2 weeks) [40]. More recently, Nomura et al. (2023) introduced an improved protocol that combines the OncoQuick^®^ density gradient tube with poly 2-methoxyethyl acrylate (PMEA)-coated plates to efficiently capture and grow colorectal CTCs [43]. Using blood samples from 41 patients with colorectal cancer, they detected CTCs or emerging tumor colonies in ~ 56% of samples after culture [43]. Spheroid-like tumor colonies or adherent clusters formed in > 50% of the CTC-positive samples within days, representing a rapid expansion of patient-derived tumor cells.

EpCAM-targeted microfluidic culture (Lipid Bilayer Device) is a microfluidic device coated with a biomimetic lipid bilayer and functionalized with anti-EpCAM antibodies that allows capturing and culturing CTCs from small blood volumes (typically 2–7.5 mL). For example, the CMx platform uses a supported lipid bilayer to gently capture EpCAM^+^ CTCs with minimal stress, followed by their release and growth in culture. Using this approach, CTCs from a patient with stage III colon cancer were cultured for at least 2 months ex vivo [41]. The released CTCs could form small colonies and even spheroids, while retaining stem-cell markers and re-attaching ability.

Short-term 3D spheroid cultures of CTCs can better mimic the tumor microenvironment. For instance, HCC CTCs have been grown as 3D micro-spheroids. In a 2021 study, CTCs from a patient with HCC were seeded in ultra-low attachment plates with specialized medium, leading to multicellular tumor spheroids that could be maintained for 12–14 days before viability declined [39]. Such 3D CTC culture system allowed investigating EMT and testing resistance to therapies (e.g., anti-angiogenic drugs) in a controlled environment that resembles in vivo tumor cell aggregates.

Size-based filtration and extended culture (MetaCell^®^) are label-independent systems to isolate CTCs based on their size and have the advantage of capturing cells without molecular bias. The MetaCell^®^ device uses an 8 μm porous polycarbonate membrane to filter blood cells/CTCs (typically 15–30 μm in diameter). Then, the membrane can be placed in culture medium, allowing the growth of the captured cells on its surface as an adherent culture. Using MetaCell^®^ or similar size-based filters, viable CTCs were obtained from ~ 8 mL blood samples of patients with esophageal, gastric, colorectal, and pancreatic cancer and maintained in culture for weeks to months [42, 44–46]. Several groups reported that some colorectal CTC populations survived and proliferated in vitro for up to ~ 6 months using membrane filtration techniques, notably from blood samples of patients with colorectal cancer. Although they are very rare cells, esophageal and pancreatic CTCs survived for weeks. This prolonged survival (far beyond the usual 1–2-week window for most CTC cultures) is critical for studying the long-term persistence of aggressive CTC subclones. It allowed the phenotypic analysis of CTCs after extended culture, revealing subpopulations with stem cell-like or mesenchymal features that might evade classical epithelial markers. Although many contaminating leukocytes are present, the approach provides a biologically rich culture to identify which CTCs survive longer.

The HCC-specific microfluidic chip is an innovative approach that targets asialoglycoprotein receptor (ASGPR), a liver-specific receptor expressed on HCC cells, to capture HCC CTCs [38]. In a study of 36 patients with advanced HCC, CTCs from 31 patients (~ 86%) could be successfully grown into spheroids after isolation using this device [38]. Higher CTC detection rates (~ 91%) were reported in complementary studies that combined ASGPR with other liver markers [74, 75]. These HCC CTC cultures were used for functional assays, such as assessing the sensitivity to liver cancer-specific drugs.

RBC lysis followed by feeder co-culture is another viable strategy in which RBCs are lysed and the remaining nucleated cells (containing CTCs) are cultured in specially conditioned media. In 2021, Yang et al. used RBC lysis to process 5 mL blood samples from 81 patients with gastrointestinal cancer (*n* = 56 colorectal and *n* = 25 gastric cancer) [50]. The pelleted cells were placed into a conditionally reprogrammed cell culture system, with a fibroblast feeder layer and a Rho-kinase inhibitor, which favors the growth of epithelial cells. After 4 weeks in culture, 38/81 samples (~ 47%) showed viable cell outgrowth and 5/38 samples formed macroscopic tumor cell colonies. Molecular characterization confirmed that at least 13 of these cell outgrowths were CTC-derived cells because they expressed telomerase (hTERT) mRNA and/or cancer-testis antigens (MAGE A1-6), which are not found in normal blood cells. These cells were subsequently used for ex vivo drug sensitivity testing, exemplifying a personalized profiling approach: clinicians could expose the patient’s CTC-derived cells to various drugs to identify the most effective treatment(s).

The GEM chip for pancreatic CTCs is an example of microfluidic devices that promote interaction between blood and capture surfaces to improve the capture of extremely low-abundance CTCs without harming their viability [47]. The GEM chip contains specially designed microchannels with ridges or grooves that create turbulent mixing, driving CTCs into contact with antibody-coated walls. Sheng et al. demonstrated that the GEM chip, coated with an anti-EpCAM antibody, could capture > 90% of tumor cells spiked in blood with high purity, and that the captured cells could be released gently (e.g., via enzymatic detachment) and remained viable. When tested using blood from patients with metastatic pancreatic cancer, the GEM chip successfully isolated CTCs in 17/18 patients (94%), a remarkably high capture rate. Furthermore, this chip allowed monitoring CTC count changes during therapy. CTC numbers correlated with the patients’ computed tomography imaging results (increase or decrease in function of the tumor burden). Therefore, the GEM chip is a powerful tool that combines efficient immunocapture and preservation of CTC viability and stem-like properties, facilitating their subsequent culture and molecular analyses.

In summary, a spectrum of short-term CTC culture techniques, from traditional density gradients and simple RBC lysis methods to sophisticated microfluidic and filtration systems, have been applied in gastrointestinal cancers. Short-term cultures (lasting one to several weeks) can be used to study CTC proliferation, sphere-forming ability, and phenotypic changes in real time. They are also useful for testing the drug responses in living CTCs, laying the groundwork for liquid biopsy-guided therapies. Maintaining CTCs in vitro remains challenging (most cultures senesce after 2–6 weeks); however, these innovations collectively improve the odds of capturing these rare, fragile tumor cells from blood and of gathering clinically relevant information within their limited lifespan.

#### Prostate cancer

In prostate cancer, two long-term CTC culture models have been obtained: the MSK-PCa1-7 series of organoids and the EMC-Pca-41 cell line [26, 27]. The MSK-PCa1-7 organoids were developed in 2014 by CTC enrichment with the RosetteSep™ technique. The EMC-Pca-41 cell line was isolated by leukapheresis followed by RosetteSep™ enrichment and then cultured in normoxic conditions. They show that it is possible to establish persistent CTC models from prostate cancer. The MSK-PCa1-7 organoids retain the genetic characteristics of the original tumor, and the EMC-Pca-41 line presents typical oncogenic alterations (TMPRSS2-ERG fusion and PTEN loss), reflecting the tumor biology of the patient from whom it was derived.

Between 2008 and 2018, many short-term prostate CTC cultures were obtained using various isolation methods. In 2009, Ficoll-Paque™ gradient centrifugation of 3 mL blood samples allowed isolating viable tumor cells in 5/8 samples. These cells could be kept alive ex vivo from 1 week to 3 months [55]. This preliminary result demonstrated the feasibility of temporary culture of prostate CTCs. In 2014, the MetaCell^®^ filtration technique allowed isolating CTCs from 8 mL of whole blood of 18/55 patients, and the captured cells could be cultured in normoxic conditions for 14 days. No direct correlation was found between the ability to culture these CTCs and the tumor T stage or the patient’s Gleason score [56].

The most recent advance (2018) combines diagnostic apheresis with EasySep™ immunomagnetic selection of EpCAM^+^ cells. This approach significantly increased the number of CTCs collected and the CTC cultures could be maintained for 4 to 6 weeks, opening new perspectives for generating more durable ex vivo models [57].

#### Lung cancer

Lung cancer is usually classified into two main categories that have distinct biological, clinical, and therapeutic characteristics: non-small cell lung cancer (NSCLC) and small cell lung cancer (SCLC). In this chapter, we describe long-term and short-term CTC lines established from patients with NSCLC or SCLC, as well as their isolation methods and conditions.

##### Non-small cell lung cancer

In 2016, Wang et al. established the first long-term NSCLC CTC line using a one-step microfluidic device combined with EpCAM- and EGFR-coated immunomagnetic beads. CTCs were initially cultured in hypoxic conditions (3% O₂) before moving to normoxia [23] This method preserved high cell viability and yielded sufficient CTCs for their multi-level genetic, protein and metabolic characterization ex vivo [23]. In 2019, Que et al. developed a second permanent NSCLC CTC line, named CTC-TJH-01, using a similar microfluidic immunomagnetic isolation strategy. This line was successfully maintained in normoxic conditions and showed tumorigenic and metastatic potential in vivo, highlighting its relevance for metastasis research [22]. In vitro, CTC-TJH-01 cells exhibit an intermediate EMT phenotype with stem-like properties and chemoresistance, and after xenografting they form tumors that colonize the lung and produce metastases [22]. Mechanistically, the altered cytokine profile (low CX3CL1 and high CXCL5 expression) in CTC-TJH-01 cells might drive their high metastatic capacity. This CTC line is a useful metastasis model for screening anti-metastatic drugs [22].

The RosetteSep™ isolation technique also has been used to enrich CTCs from blood samples of patients with NSCLC. These cells were then cultured for 20 to 50 days. Short-term CTC cultures were obtained from 9/70 blood samples (~ 13%) and their tumor-specific mutations confirmed their NSCLC origin [52].

A long-term NSCLC CTC culture was established by isolating CTCs with a specialized microfluidic chip and then culturing them at 37 °C in standard normoxic conditions [22]. This demonstrated the feasibility of establishing a permanent NSCLC CTC line that could be propagated and that exhibits tumorigenic and metastatic potential *in viv*o.

##### Small cell lung cancer

Several permanent CTC lines have been derived from blood of patients with SCLC, thanks to the higher CTC frequency in their blood samples compared with NSCLC. These CTC lines were cultured for long time in normoxic conditions and provide valuable models to study the aggressive biology of SCLC [24]. A Ficoll-Hypaque™ density gradient enrichment method is often used to isolate SCLC CTCs. For example, Klameth et al. (2017) established five permanent SCLC CTC lines from relapsed patients using Ficoll-based enrichment [24]. All lines grew spontaneously as large multicellular spheroids (tumorspheres) and expressed typical SCLC neuroendocrine markers (e.g., chromogranin A, neuron-specific enolase, synaptophysin). The tumorspheres had quiescent, hypoxic cores (KI-67^low^/CAIX^high^) and exhibited significantly greater resistance to standard SCLC chemotherapy drugs (platinum and topotecan) compared with single-cell CTCs cultured in suspension, suggesting that cluster formation contributes to chemoresistance in SCLC. In 2020, a study used the RosetteSep™ technique for CTC enrichment from 7.5 mL blood samples of patients with SCLC followed by ex vivo expansion of the captured CTCs (Liu et al. (2020). CTC expansion was successful in 18/22 samples (~ 81.8%) after 2–6 weeks of culture [53]. These cultured SCLC CTCs grew as tumorspheres and consistently expressed epithelial and neuroendocrine markers (EpCAM/CK/TTF-1/synaptophysin) and were CD45^−^, confirming their tumor origin. Importantly, the expanded CTCs remained viable (≈ 94% viability at week 2) and were used for drug sensitivity testing. Their responses to cisplatin and etoposide mirrored the patients’ clinical outcomes, underscoring the potential of short-term CTC cultures for personalized therapy decision-making and biomarker development in this aggressive cancer type. In 2023, a Ficoll-Paque enrichment method and only 6 mL of blood allowed isolating metastatic lung cancer CTCs that were maintained in culture for > 14 days, thus obtaining viable cells that could be exploited for molecular analyses [54].

#### Other cancer types

CTC cultures have been obtained also from patients with other cancer types, although the available studies concern mainly breast, lung and gastrointestinal cancers. Only one study described the isolation of the Mel-167, PEM-22, Mel-182 and PEM-78 CTC lines from blood samples of patients with melanoma using the CTC-iChip technology and their culture in hypoxic conditions [28]. Short-term CTC cultures were obtained also from patients with pleural mesothelioma: tumor cells were isolated using the MetaCell^®^ technique and maintained in normoxic conditions for 10 to 14 days [60]. Similarly, the MetaCell^®^ technique was used for isolating CTCs from blood samples of patients with urothelial tumor that were then cultured in normoxic conditions for 10 to 14 days [61]. The MetaCell^®^ technique and a porous polycarbonate membrane were used to isolate CTCs from blood samples of patients with gynecological cancers (ovarian, endometrial and cervical cancer). CTC cultures could be maintained at 37 °C with 5% CO₂ for 3 to 10 days. Several studies focused on ovarian cancer CTCs. In 2015, ovarian cancer CTCs were isolated with the MetaCell^®^ method and cultured at 37 °C with 5% CO₂ for 3 to 14 days [59] and up to 103 days [58]. In 2023, ovarian cancer CTCs were isolated using RBC lysis and cultured for 8 weeks [37]. In 2016, Kulasinghe et al. used RosetteSep™ negative selection to isolate CTCs from 25 patients with advanced-stage head and neck cancer and established short-term CTC cultures in hypoxic conditions (2% O₂, 5% CO₂). They obtained viable cultures from 7/25 samples (28%) and could maintain some of them in a 3D culture system for up to 14 days [64]. Similarly, Kuan-Chou Lin’s group obtained short-term head and neck CTC cultures (> 4 weeks) by combining Ficoll-Paque isolation and RosetteSep™ negative selection [63].

### Variability and key parameters for establishing CTC cultures

These studies show that there are many different conditions and strategies for establishing and maintaining CTC cultures, reflecting their biological complexity and the lack of a universal protocol. To better illustrate this variability, Tables 3 and 4 summarize the comparative performance of the main CTC isolation and enrichment methods. Table 3 outlines the key limitations and advantages of each capture approach and the viability of the captured cells. Table 4 presents the short-term and long-term culture success rates associated with each method. Together, these tables highlight how the viability and success rates of both short-term and long-term CTC cultures are strongly influenced by the chosen techniques.

Key parameters often include oxygen levels and whether the cells grow attached to a surface or in suspension. For short-term cultures, most laboratories use normal oxygen levels and allow the cells to attach, especially when working with tumor types the parental cells of which naturally grow adherent. Conversely, for long-term culture, many laboratories prefer low-oxygen conditions and growth in suspension because this tends to promote CTC survival and helps to maintain their stem cell-like state, which is often linked to metastasis and therapy resistance [21, 24, 76]. The culture medium is another important variable. The most common formulations are RPMI-1640, DMEM, DMEM/F12, and Advanced DMEM/F12; all provide the basic nutrients required for mammalian cell growth. However, Advanced DMEM/F12 contains additional proteins, such as AlbuMAX II, human transferrin and insulin, that can improve cell viability and proliferation. Nevertheless, supplements and adjustments vary widely among studies, making it difficult to propose a standardized approach for culturing CTCs from different cancer types.

Overall, the literature shows that there is no single ideal method or fixed condition for CTC culture, and success rates depend on the complex interplay between technical parameters and intrinsic biological factors, such as cancer (sub)type, metastatic status, intra-patient heterogeneity, and even timing of blood collection relative to treatment. This variability explains why same protocols can yield very different outcomes from one patient to another. In the past decade, technical advances in enrichment methods (moving from EpCAM-only capture to multiparametric or physical approaches), culture conditions (specialized media, 3D formats, co-culture, transient hypoxia), and blood sampling volume (from standard blood tubes to leukapheresis) have progressively improved the chances of establishing (short-term or extended) CTC cultures, transforming them from a rare occurrence to a relatively more frequent achievement.

Nevertheless, even with the best techniques, not all tumors release cells that can grow ex vivo. Inter-patient heterogeneity and CTC biological variability remain the main determinants. Some CTCs possess stem cell and adaptability features (stress resistance, high metastatic potential) that allow them to proliferate in culture, while others enter apoptosis or dormancy and will never grow in culture, despite the efforts. This explains why despite the technical progress, permanent CTC lines remain few and difficult to obtain.

**Table 3:** CTC enrichment techniques: capture efficiency and cell viability

	Enrichment method	Capture efficiency	Cell viability
Biological properties	Positive selection	High CTC purity when the target antigen is expressed, but misses antigen-negative CTCs. Recovery ~ 50–85% in spiking assays; binding sites can saturate at high CTC loads. Low false-positive rate (non-CTCs rarely bind). May activate some cellular pathways, thereby modifying the cell characteristics before downstream analyses.	Lower viability than other methods: antibody/bead attachment can impair cell survival or proliferation; some platforms require fixation, preventing the culture of enriched CTCs.
	Negative selection	Moderate purity: removes other cells instead of capturing CTCs, enabling the recovery of all CTC subpopulations. Large blood volumes, including whole-blood processing and leukapheresis products, can be used to substantially increase the total CTC yield. Trade-off: lower initial specificity, with residual non-tumor cells, requiring downstream CTC identification. Negative selection maximizes CTC coverage, but reduces purity. Minimal phenotype bias: does not rely on a specific tumor marker, ensuring the non-discriminatory capture and low risk of missing CTC subsets.	Excellent viability: as CTCs are untagged and unfixed, removing the other cell types leaves them intact and highly viable (often > 90% with label-free methods). Low manipulation stress: no harsh chemical or physical force applied to CTCs. Caveat: co-enriched leukocytes may compete with CTCs in culture or secrete factors that can hinder growth if not removed.
Physical properties	Size-based, density or deformability approaches	Moderate to high efficiency (method-dependent): many CTCs are larger and less deformable than leukocytes, allowing good capture yields. Filtration and inertial microfluidic systems typically give ~ 78–90% recovery under optimal laboratory conditions. Limitations: small or highly deformable CTCs are often lost and large leukocytes can be captured (megakaryocytes). Pore clogging may occur in samples with high cell or debris content, reducing both efficiency and purity. Marker-independent (label-free): does not rely on surface markers, enabling the capture of both epithelial and mesenchymal CTCs without immunological bias.	Generally good viability: filtration is gentle and label-free, and recent approaches reduce shear stress. Viability is often 70–98%, depending on the device and flow conditions. Cells can be recovered alive: the captured CTCs are usually retrievable for culture, and newer systems avoid pre-fixation, thus preserving cell viability for downstream growth.

**Table 4:** CTC isolation methods: culture success rate

	CTC isolation method	Short-term culture success (few weeks)	Long-term culture success (stable cell line for > 6 months)
Biological properties	Positive selection	Low: a viable CTC culture for more than 1 week is derived from ~ 10–15% of samples. Direct immunocapture can damage CTCs, making sustained growth rare. Most studies report only a small fraction of successful short-term cultures when using purely positive selection.	Near zero: typically, < 5% of cases yield a stable long-term CTC line. Long-term CTC lines generated through positive selection are extremely rare, because the process is considered too harsh on CTCs, resulting in a negligible long-term success rate.
	Negative selection	Moderate: in favorable conditions, a short-term CTC culture is derived, on average, from ~ 40–50% of patient samples. Negative selection methods better preserve CTC viability, thus increasing the initial culture success. For example, a RosetteSep-based study reported short-term cultures from 16 of 27 samples (~ 59%) [77].	Low: only few samples (< 10%) give rise to a true long-term CTC line. However, negative selection has produced more established CTC lines than other methods and is behind many of the rare success stories. Still, the overall long-term success rate remains very low, generally < 10%.
Physical properties	Size-based, density or deformability approaches	High (initially): short-term culture success is often high after physical enrichment. For example, Ficoll density gradients enabled short-term CTC cultures from 100% of 12 samples (all maintained for > 30 days) [78]. A filter-based system (MetaCell^®^) reported ~ 65% short-term success. However, many of these cultures do not progress beyond few weeks, often dying or stagnating after the initial growth [34].	Very low: only ~ 0–5% of samples yield a durable long-term CTC line. In practice, establishing permanent CTC lines using only physical enrichment is rare. Few SCLC CTC lines were generated using density-gradient methods, but these are exceptions with very low efficiency.

### Establishment of CTC-derived xenograft models

**Fig. 2:**
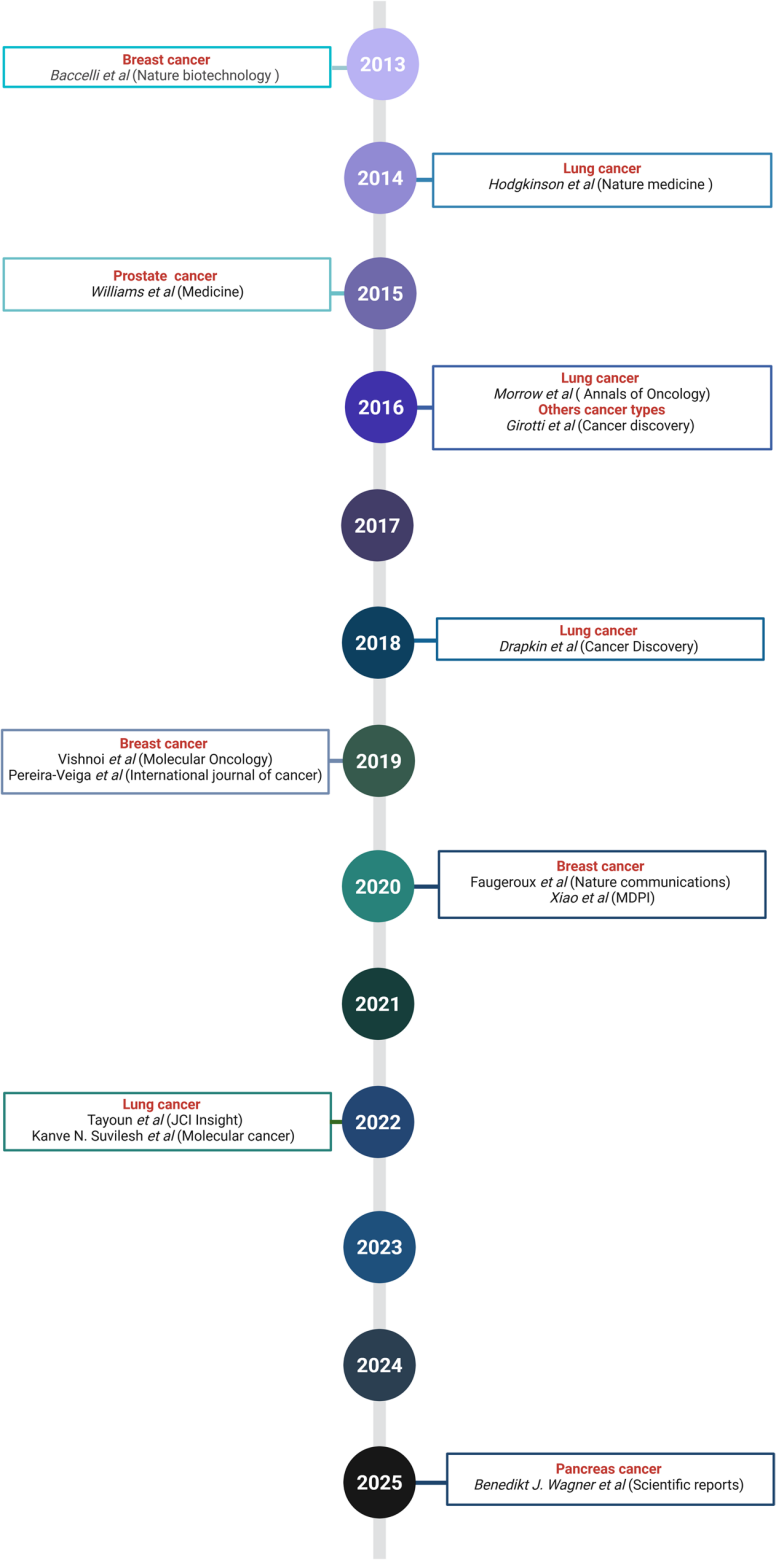
Timeline of CTC-derived xenograft (CDX) model development by cancer type*.* Each point represents the first publication reporting the generation of a CDX model. The cancer type is indicated next to each point, as well as the first author and the journal name

In patient-derived xenografts (PDX), human tumor samples are xenografted into immunodeficient mice. These models are considered the most reliable proof of the stem cell properties of tumor cells and have one of the best predictive values among preclinical models. However, obtaining biopsies from the primary tumor and its metastases can be invasive and complex, and PDX models still fail to fully capture the original tumor heterogeneity. These difficulties might be overcome by using CTCs, which represent a more aggressive subpopulation of tumor cells. Moreover, blood sampling is non-invasive compared with tissue biopsies. CDX models constitute a molecularly faithful snapshot of the disease and they can act as surrogates of otherwise inaccessible metastatic tissues. Table 5 lists the studies on CDX according to the tumor type. Figure 2 illustrates the chronological timeline of CDX development in different cancer types, providing an overview of how these models have emerged and evolved over time.

**Table 5:** List of the characteristics of the available CDX mouse models

Cancer type		CTC culture period	Year	Success rate	CTC enrichment and isolation	Blood volume	Mouse strain	Xenografts	Ref
Breast cancer		/	2019	1/3	FACS sorting (CD45^−^/CD34^−^/CD105^−^/CD90^−^/CD73^−^ cells)	8 mL	NSG	Intracardiac	[79]
		6–15 months	2013	6/110	Hematopoietic cell depletion	7.5 mL		Femoral tumor cavity	[80]
		5 months	2019	1	Density gradient centrifugation (Histopaque) in SepMate™ tubes	7.5 mL	NMRI-Foxn1nu/nu (Nude)	Subcutaneous	[81]
Lung cancer	Lung cancer	Up to 1 year/2.4–4.4 months	2020	17%	RosetteSep™	10 mL	NSG	Subcutaneous in flanks	[82]
	Non-small cell lung cancer	95 days	2016	1		10 mL			[83]
		100, 200, 116, 100 days	2022	4/55		7.5 mL		Interscapular fat pad	[84]
		/	2022	2/10	/	/	NSG	Subcutaneous	[85]
	Small cell lung cancer	115 days	2018	16/42	CTC-iChip device	15–20 mL	NSGN3, N9H1N3, NN3, N14	Subcutaneous	[86]
		4 months	2014	6/20	RosetteSep™ Human Circulating Epithelial Tumor Cell Cocktail	10 mL	NSG	Subcutaneously in flanks	[87]
Prostate cancer		10 months	2020	0/15	RosetteSep™ CTC enrichment cocktail containing an anti-CD36™ antibody	7.5 mL	NSG	Interscapular region	[88]
		165 days	2020	1/7	Diagnostic leukapheresis (DLA) + RosetteSep™ CTC enrichment cocktail containing an anti-CD36™ antibody	2/3 of DLA		Interscapular region	[88]
Melanoma		1 month/2.5 months	2016	6/47	RosetteSep™ depletion cocktail	7.5 mL	NSG	Subcutaneous	[89]
Pancreatic cancer		/	2024	/	EpCAM-coated beads	5 mL	/	Spleen	[90]

#### Breast cancer

In 2013, Baccelli et al. developed an in vivo test to study CTCs in a mouse model [80]. For the first time, they injected CTCs from patients with metastatic breast cancer, isolated through hematopoietic cell depletion, in the medullary cavity of the femur of immunodeficient mice. This led to the development of bone, lung and liver metastases. CDXs were only formed in mice when a large number of CTCs could be isolated from the patient blood samples. The number of CD44^+^, CD47^+^ and MET^+^ CTCs correlated with reduced progression-free survival in these patients.

The first triple-negative breast cancer (TNBC) CDX was obtained from CTCs of patients who had > 900 CTCs in 7.5 mL of blood, as estimated using the CellSearch^®^ system based on EpCAM-positive enrichment [81]. These CTCs were isolated using a density gradient centrifugation protocol (Histopaque) in SepMate™ tubes. The isolated CTCs were transplanted subcutaneously into mice, and tumors were detected after several months. Tissue samples from metastatic sites were also collected concomitantly with CTCs, allowing the real-time study of tumor samples. Vishnoi and colleagues injected in immunodeficient mice CTCs from patients with TNBC isolated by FACS. They showed that the distinct transcriptomic signatures of CTCs were preserved in the CDX model [79]. They highlighted a pathological signature associated with CTCs in liver metastases, providing valuable insights into the mechanisms of TNBC recurrence in the liver. In 2020, Klotz et al. established a CDX model to study brain metastases of luminal breast cancer [91]. They used four GFP-Luc-labeled CTC lines, previously isolated from patients with luminal breast cancer, and monitored metastasis development in mice for at least 5 months to identify genes involved in brain metastases.

#### Prostate cancer

In 2020, Faugeroux et al. were the first to generate a CDX model for prostate cancer in which the time required for tumor development was greatly reduced [88]. They collected ~ 20,000 CTCs by leukapheresis followed by hematopoietic cell depletion to facilitate CDX derivation. In the primary tumor, they detected neuroendocrine markers the expression of which was increased in the CDX. This indicates the emergence of a new androgen receptor-negative and neuroendocrine-positive phenotype in the xenografts. The CDX model also accurately recapitulated the patient’s response to docetaxel and enzalutamide. Moreover, a CDX-derived CTC line showed similar phenotypic, functional and genetic traits as the original CDX. It has been invaluable for testing new treatments. The model has provided insights into the progressive acquisition of neuroendocrine transdifferentiation, offering an exciting and effective tool for drug evaluation.

#### Melanoma

Few studies focused on the development of CDXs for melanoma. In 2016, CTCs were isolated with the RosetteSep™ technique from a blood sample of a patient with an ultra-aggressive, therapy-resistant BRAF-V600E melanoma and then used to create a CDX model [89]. The CDX showed histological features similar to those of the original melanoma, and mice xenografted with this CDX developed lung micro-metastases. After removal of the primary tumor, mice developed many liver metastases, mirroring the metastatic progression observed in the patient. However, the CDX demonstrated an almost complete response to the treatments that had been ineffective in the patient. In 2018, Vishnoi et al. isolated CTCs from blood samples of patients with melanoma using flow cytometry and generated CDX models by intracardiac injection of these CTCs in mice [92]. In the xenografted mice, tumor cells disseminated in the long bones and in typical target organs. After 6 months, routine immunohistochemistry evaluation revealed no visible metastasis. However, immunostaining with specific markers detected the presence of human melanoma cells in various organs.

#### Lung cancer

Since 2014, several studies have shown that it is possible and useful to develop CDXs to study and treat lung cancer. Hodgkinson et al. proved that in patients with advanced metastatic SCLC, CTCs isolated in sufficient numbers (> 410 cells) could form tumors after subcutaneous injection in NOD/SCID immunodeficient mice, leading to CDX creation in 6/20 cases [87]. In this protocol, CTCs were obtained by immunomagnetic isolation targeting EpCAM, demonstrating that a blood sample is a usable source of tumor cells to reproduce the disease in mice. Two years later, Morrow et al. [83] extended this observation to NSCLC, reporting the derivation of a CDX from CTCs with a mesenchymal phenotype (EpCAM- negative). This showed that the absence of EpCAM⁺ CTCs does not prevent tumor dissemination. Here, CTCs were isolated using a label-free method based on the cell physical properties, illustrating the importance of diversifying capture approaches to include non-epithelial CTC populations. Then, Drapkin et al. [86] optimized the process by using an automated microfluidic chip (Herringbone Microfluidic Chip) to isolate viable CTCs from blood samples of patients with SCLC. Upon injection into NSG mice, sometimes in the presence of Matrigel to promote tumor take, these CTCs produced CDXs in 38% of cases (vs. 89% for PDX from solid tumors). Transcriptomic analyses revealed a marked MYC signature and treatment resistance, faithfully reproducing the responses observed in patients. Lallo et al. [93]. confirmed that CDXs can accurately reflect the genetic and phenotypic characteristics of the original tumors. They used these models to evaluate the efficacy of targeted treatments, such as the PARP inhibitor olaparib and the WEE1 inhibitor AZD1775, and found that PALB2 mutations could be associated with therapeutic resistance. Two studies that thoroughly characterized CDXs [94, 95] highlighted the strong SCLC heterogeneity: CDX success rate after subcutaneous injection of isolated CTCs did not exceed 17%, and four main molecular subtypes (ASCL1, NEUROD1, POU2F3, ATOH) were identified. To better understand intratumoral complexity, Stewart et al. [96]. analyzed CDXs by single-cell RNA-sequencing and demonstrated the coexistence of multiple resistance pathways within distinct subpopulations. For NSCLC, Tayoun et al. [84] showed that CDXs could be obtained by implanting enriched CTC fractions (from 35 to 3,500 cells, isolated by microfluidics and FACS) in the interscapular fat pad of NSG mice. They found that xenograft success depended on the number of CTCs injected and also on their quality and phenotype.

Lastly, Suvilesh et al.. (2022) proposed a two-step approach: first they established a PDX from surgical samples of non-metastatic patients, and then they isolated CTCs from these PDX by density gradient centrifugation with leukocyte depletion, before subcutaneous injection (with or without Matrigel) into new NSG mice [85]. Among the ten PDX lines, two allowed the establishment of stable CDXs (20% of success). These models, derived from genuine liquid biopsies, reproduce the clinically undetectable residual disease and offer a unique platform for studying metastatic progression in a curative context. Single-cell transcriptomic analysis of these CDX revealed the emergence of a regenerative type II alveolar pneumocyte population, similar to that observed in human lung metastases, highlighting the impact of tumor cells passing through the circulation. These models have also been used to test carboplatin/paclitaxel with variable responses; in some cases, MYC overexpression–associated chemoresistance could be overcome in vivo by inhibiting this pathway.

#### Pancreatic cancer

In a recent study, CTCs were isolated with beads coated with the universal cancer-binding protein VAR2 from patients with locally advanced or metastatic pancreatic ductal carcinoma to generate tumor organoids and xenografts in mice. A median of 65 CTCs per 5 mL blood sample were detected and ~ 9% of CTCs expressed CXCR4. These CTCs had a circulating cancer stem cell phenotype characterized by stem-like properties and exacerbated metabolism. From these CTCs, 3D organoid-type cultures were obtained in 2 to 4 weeks that were then propagated into adherent cell lines over 6 weeks. Cells from these CTC cultures were used to generate highly tumorigenic and metastatic in vivo models. When injected in mice (splenic route with liver relay), they generated more liver metastases than cells derived from classical PDX, confirming their increased aggressiveness. This study also showed that the success of organoid establishment correlated with the initial CTC number, and more specifically with the presence of CXCR4^+^ CTCs. Moreover, in these CTC-derived models, a compound screen targeting “stemness” pathways revealed that inhibiting the stearoyl-CoA desaturase (SCD1) enzyme induces a promising antitumor response [90].

Different parameters may influence the success of establishing CTC lines with a more or less long culture periods.

**Table:** Box 1: Limitations and challenges of CTC cultures

CTC sampling and analytical processes	Culture conditions
Blood volume	Medium composition
Sampling site	Culture support
Cancer type	Co-culture
Tumor stage	Hypoxia/normoxia
Enrichment techniques	

**Table:** Box 2: Advantages and disadvantages of CTC models

Model	Advantages	Disadvantages
In vitro CTC culture	• Personalized medicine with patient-specific CTC lines and drug sensitivity testing • Understanding the biology of metastasis development • Biomarker discovery • Low cost	• Lack of model complexity
CDX	• Representation of human tumors *via* CTC transplantation and preserved characteristics • Drug testing and development: clinical response prediction • Resistance mechanism identification • Biomarker validation • Translational research: bridge between research and clinic	• High cost • Ethical considerations • Inter-animal variability • Immunodeficient mice

**Fig. 3:**
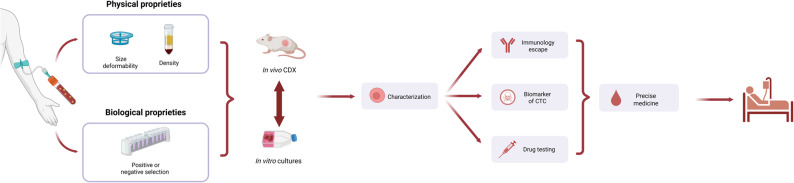
Establishment and use of preclinical in vivo/in vitro CTC models. To establish preclinical in vivo and in vitro models, viable CTCs are enriched from peripheral whole blood by exploiting their physical (e.g., filtration, density gradient centrifugation) or biological properties (e.g., positive or negative depletion, microfluidic devices). The isolated CTCs are cultured in vitro to establish CTC lines (in suspension or spheroids). In vitro cultures can be used to generate CTC-derived xenografts (CDXs) in vivo, and CDXs can provide material for establishing in vitro cultures. These two model types are valuable sources for subsequent single-cell analyses of CTCs (mutation analysis, RNA sequencing, mass cytometry, microscopy). They facilitate the characterization of metastasis-competent CTCs, the discovery of new biomarkers, understanding immune evasion mechanisms, and drug testing. These advancements contribute to more personalized medicine *Created with BioRender.com*

. In summary, CTC lines and CDX models offer powerful tools for advancing cancer research, improving our understanding of tumor biology, and enhancing the development of personalized and effective cancer therapies. Figure 3 provides a schematic overview of the key methodological steps and experimental strategies used to establish CTC-derived cell lines and CDX models.

### Establishment of DTC lines from solid cancers

DTCs are mainly found in bone marrow and represent a key cell population in the metastatic process. DTCs proliferate very little, and they can persist in a dormant state for years before eventually giving rise to overt metastases. The functional study of DTCs has long been limited by their rarity and poor growth in vitro. Pantel’s group was the first to develop a protocol for isolating and immortalizing human DTCs from the bone marrow of patients with cancer [97]. In this approach, bone marrow aspirates are cultured on an extracellular matrix substrate in medium enriched with EGF and bFGF to stimulate epithelial tumor cell growth, and then cells are immortalized by microinjection of the SV40 large T-antigen gene. This method allowed establishing permanent DTC lines that express epithelial markers, such as cytokeratins, confirming their carcinoma origin. Remarkably, some of these cell lines recolonized the bone marrow when transplanted in immunodeficient SCID mice, demonstrating that they retain bone marrow tropism and metastatic behavior in vivo [97]. These DTC lines are a unique resource to study cancer cell dormancy, therapy resistance and metastatic reactivation in a variety of cancer types. Table 6 lists the studies on DTC according to the tumor type.

**Table 6:** List of the existing DTC lines

Tumor origin	Cell line	Biological source	Isolation/Culture method	Yield/Viability	Ref
Breast	BC-M1	Patient bone marrow	ECM-based culture, EGF and bFGF supplementation → SV40 large T-antigen immortalization	Very low initial yield (few DTCs); stable cell line after 4–6 weeks	[97]
	BC-H1			Few initial cells; very limited expansion	[97]
	BC-K1/BC-P1/BC-S1			Extremely low initial yield; immortalization achieved from single-cell clones	[97]
Prostate	PC-H1/PC-R1/PC-S1			Very low yield, but gave rise to long-term cell lines	[97]
Colon	CC-B1			Few initial tumor cells; immortalized into a stable cell line	[97]
Lung	LC-M1			Few initial cells; established as one stable cell line	[97]
	SBC-3/SBC-5/SBC-7	Pleural/pericardial effusions; bone marrow metastases	Direct suspension culture in RPMI with fetal bovine serum (+ IL-2 for SCLC) (no viral immortalization)	High yield (10^5^–10^6^ cells from patient samples); stable continuous cells lines	[98]

#### Breast cancer

The BC-M1 cancer cell line is the most extensively studied breast cancer DTC model to date. It was established from DTCs isolated from the bone marrow of a patient with breast carcinoma, using the SV40-based protocol described by Pantel et al. [97, 99]. BC-M1 cells retain a partially epithelial phenotype: they co-express epithelial markers (cytokeratins, EpCAM) and the mesenchymal marker vimentin, indicating incomplete EMT and substantial phenotypic plasticity. Subsequent studies revealed several notable adaptive traits of the BC-M1 line. Indeed, these cells exhibit chronic activation of the unfolded protein response through the chaperone GRP-78/BiP. This confers resistance to metabolic stress and promotes the formation of filamentous cytoplasmic structures that enhance cell survival in hostile conditions. Additionally, in hypoxic conditions, the immune checkpoint protein PD-L1 is upregulated in BC-M1 cells. This effect is reversible upon reoxygenation, suggesting a dynamic, microenvironment-dependent immune evasion mechanism [100].

Patel’s group also established several other breast cancer DTC lines (BC-H1, BC-K1, BC-P1, and BC-S1) using a similar SV40 immortalization protocol [97]. Like BC-M1 cells, all these cell lines express cytokeratins (reflecting their epithelial origin), but display varying degrees of phenotypic plasticity between epithelial and mesenchymal states. Comparative proteomic analyses of BC-H1, -K1, -P1, and -S1 cells revealed considerable inter-cell line heterogeneity in the expression of proteins involved in the cytoskeleton, stress signaling pathways, and EMT regulation. This suggests that each cell line has distinct adaptive capacities, depending on its cellular context [101]. However, unlike BC-M1 cells, these other breast cancer DTC lines remain only partially characterized. Their specific mechanisms of metabolic stress resistance and immune evasion have not been thoroughly explored. Nevertheless, these DTC lines broaden the experimental panel of breast-cancer derived DTC models. Importantly, they also provide a valuable platform to study cancer dormancy, a poorly understood yet clinically critical phenomenon [5].

Researchers have also employed PDX models to study the dissemination of breast cancer cells to bone marrow in vivo. Pillai et al. developed innovative experimental systems where breast tumor PDX fragments implanted in NOD/SCID mice gave rise to spontaneous DTCs in the mouse bone marrow [102]. Using a combination of sensitive RT-qPCR and global transcriptomic analysis (microarrays), they detected human breast cancer DTCs in the murine bone marrow and showed that in these cells, epithelial markers (e.g. *KRT19*) were downregulated and several EMT-associated transcription factors (e.g. *SNAI1*, *GSC*, and *FOXC2*) were upregulated. Comparative profiling led to the identification of a 16-gene “BM-DTC signature” that included *ALCAM*, *CD44* and *MALAT1* and that distinguished normal bone marrow from bone marrow with micro-metastases, highlighting the potential prognostic value of this DTC-specific signature [102]. The same group used a microfluidic enrichment technology (the Parsortix^®^ microfluidic device, originally designed to capture CTCs) to isolate DTCs from clinical bone marrow aspirates [103]. Single-cell transcriptomic profiling of the enriched cells revealed the coexistence of EpCAM^+^ (epithelial-like) and EpCAM^−^ (mesenchymal-like) DTC subpopulations in the bone marrow of the same patient, reinforcing the concept of EMT-driven phenotypic heterogeneity in DTCs. Together, these complementary approaches (long-term DTC lines and in vivo PDX-based models) provide powerful platforms to study DTC biology in breast cancer. They allow researchers to explore DTC heterogeneity, transitions between dormancy and reactivation, metabolic adaptations to the bone marrow niche, immune evasion strategies, and other mechanisms underlying bone marrow colonization and metastatic progression.

#### Prostate cancer

Using similar methodologies, researchers have established prostate cancer DTC lines from bone marrow. The pioneering works by Pantel et al. (1995) and Offner et al. (1999) led to the derivation of four prostate DTC lines (PC-E1, PC-H1, PC-R1, and PC-S1) from bone marrow aspirates of patients with metastatic prostate cancer [97, 99]. These rare disseminated cells were immortalized with the SV40 large T-antigen, as done for the breast cancer DTC lines. Prostate DTC lines proliferate more slowly (~ 10-fold) than conventional metastatic prostate cell lines, suggesting that they remain largely dormant (likely arrested in the G₀/G₁ phase of the cell cycle) in culture.

Studies using these dormant prostate DTC lines have yielded important insights. For example, Offner et al. (1999) observed that DTCs often lack mutations in the p53 tumor suppressor gene, even when the corresponding primary prostate tumor harbors a p53 mutation [99]. This implies that prostate cancer cell dissemination can occur at an early stage of tumor development, potentially before the accumulation of genetic alterations (e.g. p53 mutation) in the primary tumor. Moreover, prostate DTCs specifically interact with the bone marrow microenvironment. Particularly, they can “roll” along the bone marrow endothelium by binding to E-selectin molecules expressed on endothelial cells *via* the sialyl Lewis X ligands expressed on their surface. This rolling adhesion mechanism might facilitate DTC lodging and colonization of bone marrow niches, thereby promoting metastatic outgrowth in bone [104]. Integrins expressed on DTCs, such as β1 and αVβ3 integrins, also have been implicated in this bone-homing process [105]. These interactions with E-selectin and integrins highlight the specialized adaptations that allow prostate DTCs to seed and persist in the bone marrow.

#### Lung cancer

Following the approach first used for breast cancer, the LC-M1 cancer cell line was generated using DTCs isolated from the bone marrow of a patient with lung cancer [97]. Although the LC-M1 lines comes from a NSCLC, it shows striking similarities to the BC-M1 cell line derived from breast cancer DTCs. Both cancer cell lines display a strong and persistent activation of the unfolded protein response, suggesting shared adaptive mechanisms. Because of these features, LC-M1 has become a useful cross-organ model to study DTCs from breast, prostate, or lung cancer, and to identify molecular signatures linked to therapy resistance, phenotypic plasticity, and metastatic dormancy [100].

In SCLC, several cell lines have been derived from metastatic sites, such as bone marrow and pleural or pericardial effusions. Among them, the SBC-3 to SBC-7 cell lines were established by Kishimoto et al. in the 1980 s, using tumor cells taken from various metastatic locations, including bone marrow, pleural/pericardial fluids and bone lesions [98]. Their establishment did not involve viral immortalization: metastatic tumor cells were simply cultured in standard medium (RPMI with fetal bovine serum, sometimes supplemented with IL-2 for SCLC). Only the cells capable of growing on their own in the long term survived and formed stable cell lines.

Some of these DTC lines have been particularly well studied. The SBC-3 cell line, derived from a bone marrow aspirate of an untreated patient with SCLC, and the SBC-5 line, isolated from a malignant pleural effusion, were used to create reproducible in vivo models. For example, intravenous injection of SBC-5 cells in mice consistently produces bone metastases. This model contributed to demonstrate the key role of parathyroid hormone–related protein (PTHrP) in bone colonization: treating mice with neutralizing antibodies against PTHrP significantly reduced osteolytic lesions, showing its importance in SCLC bone metastasis [106].

Although they were derived using cells from metastatic sites, the SBC cell lines stably express typical neuroendocrine markers, such as CD56/NCAM, synaptophysin, and chromogranin A, with some variations among subclones or depending on the culture conditions [107] This stable expression of neuroendocrine markers is particularly valuable, as it ensures that these models faithfully recapitulate the biology of classical SCLC and remain relevant for studying tumor behavior and therapeutic vulnerabilities. Subsequently cancer cell lines, such as SBC-6 (pleural effusion) and SBC-7 (pericardial effusion), show strong phenotypic plasticity and can spread to multiple organs in experimental models [108].

Lastly, recent molecular analyses led to the reclassification of some of these historically SCLC-labeled cell lines. Notably, SBC-5 shows transcriptomic and genomic features that are more consistent with SMARCA4-deficient thoracic tumors (SMARCA4-UT) than with classical SCLC [109]. This reclassification should be considered when interpreting data obtained from SBC-5 and similar cells lines because their biology differs from that of true SCLCs.

#### Digestive cancer

Efforts to establish DTC-derived models have extended to colorectal cancer. A notable example is the CC-B1 cell line that was derived from bone marrow DTCs of a patients with colorectal carcinoma via SV40 large T-antigen immortalization. Using the same protocol as described for breast cancer, the CC-B1 cell line was generated as a permanent line from micro-metastatic tumor cells without significantly altering their epithelial characteristics [97]. Consistent with its epithelial origin, CC-B1 cells express classical epithelial markers, such as various cytokeratin types and E-cadherin, but not hematopoietic cell markers, confirming that they are epithelial tumor cells rather than contaminating blood cells. Putz et al. (1999) characterized several DTC lines (including CC-B1) and demonstrated that this epithelial phenotype is maintained despite the SV40-induced immortalization. Such micro-metastatic cell models (e.g. CC-B1) are unique tools for examining how DTCs persist in a dormant state, how they exhibit cellular plasticity, and in what conditions they are reactivated [110].

DTC-derived models, obtained directly from the patients’ bone marrow (such as BC-M1, PC-E1, LC-M1) or developed through innovative PDX-based approaches, offer a unique window into the biology of occult micro-metastases. These models recapitulate many key features observed in vivo, including the pronounced phenotypic plasticity, activation of stress resistance pathways, and capacity to remain quiescent for extended periods, while allowing controlled experimental studies of DTC interactions with the bone marrow niche. Despite their scientific value, most DTC-derived cell lines (apart from well-studied examples, such as BC-M1 and PC-E1) remain poorly characterized. Moreover, for some tumor types, such as NSCLC and colorectal cancer, established DTC lines are lacking, underscoring the need of new model systems. Future efforts that combine advanced co-culture techniques (to better recapitulate supportive stromal niches), microfluidic “organ-on-chip” systems, and refined in vivo PDX models will be crucial to better study early tumor cell dissemination and to unravel the mechanisms by which dormant DTCs reawaken and drive metastatic relapse.

###  Clinical potential of CTC lines and CDX models

The use of CTC models and their thorough characterization have led to many significant discoveries that have improved our understanding of the metastatic cascade and the nature of metastases. Specifically, CTCs do not exhibit a purely mesenchymal or epithelial phenotype, but rather an intermediate [17, 18] phenotype that contributes to their aggressiveness and plays a key role in tumor progression [111–113]. Moreover, CTC lines can induce endothelial cell tube formation in vitro through the secretion of angiogenic factors, highlighting their potential to promote blood vessel formation, which is essential for tumor growth and dissemination.

Other studies showed that CTCs can display phenotypic and functional characteristics of cancer stem cells, including ALDH expression [17]. Moreover, the CTC-ITB-01 cell line revealed a population of interchangeable adherent and non-adherent cells, reminiscent of cancer stem cell behavior [114]. Transcriptomic analyses showed that CTCs and CDXs share a common origin with the patients’ tumors, and increased regulation of the WNT pathway, which is associated with higher risk of metastasis [81, 115]. Several genes, such as *AURKB*, *HIST1H4A1*, *MELK* and *PCDHA8*, have been identified as potential indicators of tumor dissemination. These genes play crucial roles in cell cycle regulation, proliferation, apoptosis, and tumor progression. Moreover, in a colon cancer CTC line (CTC-MCC-41), genes involved in energy metabolism (*PPARGC1A*, *PPARGC1B*, *FABP1*, *ALDH3A1*), DNA repair (*BRIP1*, *FANCB*, *FANCM*), and stem-like properties (*GLS2*, *CBS*, *CTH*) are differentially expressed. This highlights CTC role in colorectal cancer progression, emphasizing their potential as specific biomarkers [7]. It also opens the door to the development of new therapies targeting the stem-like properties of CTCs, which are responsible for metastasis and tumor relapse in patients with colon cancer.

Interestingly, in the CTC-MCC-41 cell line, genes involved in fatty acid metabolism are upregulated, particularly *PPARGC1A* and *PPARGC1B* that are essential for converting glucose into fatty acids to support tumor growth [7]. This cell line molecular signature includes many mitochondrial genes, emphasizing the connection with energy production and apoptotic balance. Anti-apoptotic genes (e.g., *BCL2A1*) and pro-apoptotic genes (e.g., *BIK* and *BAX*) are critical for CTC survival. Moreover, ABC transporters, such as ABCB1, ABCA12, and ABCC2, are highly upregulated, contributing to colorectal cancer initiation and progression [7].

Most studies focused on the molecular characterization of these cell lines to describe their mutation status or to identify *de novo* mutations in CTCs. This is useful to develop targeted approaches in this population. The goal is to identify drug-resistant mutations or mechanisms of resistance to specifically tailor treatments. Yu et al. established CTC lines and revealed that they acquired *de novo* mutations during treatment. Moreover, drugs targeting these mutations were successfully tested in vitro in these CTC lines [14].

CDXs, generated at different stages from the same patient, can accurately reproduce the drug sensitivity changes during disease progression, highlighting the translational potential of this strategy. SCLC CDXs faithfully replicate the donor patient’s responses to platinum and etoposide, enabling clinically relevant studies on SCLC biology and providing patient-derived models to test targeted therapies. These data formally demonstrate that CTCs are tumorigenic and that the tumors they form replicate the donor patients’ tumor biology, confirming CTC importance in disease progression [87]. Koch et al. provided additional evidence of the functional relevance of CTC-derived models. They showed that the CTC-ITB-01 cell line exhibits tumorigenic behavior in mouse models and brought insights into CTC plasticity and ability to replicate the donors’ tumor phenotypes, including the response to treatments. Notably, they used this cell line for testing the sensitivity to CDK4/6 inhibitors, such as palbociclib, demonstrating its clinical potential to guide therapeutic decision-making [17].

Several studies showed that short-term CTC cultures can reflect the patient’s disease status. A large study used peripheral blood mononuclear cells isolated from blood samples of 5,509 patients (25 cancer types) and 10,625 healthy people to detect circulating ensembles of tumor-associated cells (C-ETAC). After five days of culture, the formation of C-ETACs, which include clusters of CTCs and associated immune cells, reliably indicated the presence of cancer [116]. Another study showed that the culture period of CTCs isolated from blood samples of patients with breast cancer could predict survival. Specifically, CTC cultures that lasted > 23 days were linked to shorter progression-free survival (*p* = 0.008), but not overall survival (*p* = 0.01) [117].

In addition, many CTC lines show strong tumor-forming abilities, significantly advancing our understanding of cancer metastasis mechanisms using combined in vitro and ex vivo studies [18]. Yet, the specific CTC biological features and metastatic mechanisms remain largely unknown. The existing CTC lines offer valuable tools for more studies on iron metabolism pathways and DNA methylation patterns, all crucial for understanding CTC-mediated metastasis [28, 118]. Besides their molecular characterization, CTC and CDX models can also unveil dynamic mechanisms of cancer evolution.

In conclusion, all these findings, derived from multiple models, highlight the value of CTC lines and CDX models as powerful tools to study tumor progression, treatment response, and the biology of metastasis-initiating cells. Together, they underscore the growing interest in CTC-based models for translational research and their unique potential to advance personalized medicine by capturing patient-specific tumor dynamics. By providing an ex vivo model to understand how these aggressive cells adapt, survive and spread, CTC lines and CDX models offer promising opportunities to develop more precise and effective therapeutic strategies tailored to the individual patient characteristics, paving the way for future innovations in cancer treatment.

### Future prospects for CTC, CDX and DTC models in cancer research

CTC models and DTC have already proven their importance. They also represent fertile ground for future research to broadening their scope, improving techniques, or deepening our understanding of biological mechanisms. These research efforts will further transform our approach to fighting cancer. Indeed, recent advances have enabled the establishment of rare preclinical models, including a handful of stable CTC lines and CDX models, that offer a direct window into human metastasis. These models also allow scientists to track, in near real time, the patient’s tumor evolution under treatment pressure; however, their successful creation remains extremely challenging due to CTC scarcity and fragility.

First, there is room for improvement in making these models more representative. Some cancer types have not been included yet, limiting their applicability across all pathologies. Even for the already studied cancer types, only a small number of permanent CTC lines have been successfully established, typically from patients with very advanced disease and high CTC counts, underscoring how difficult it is to capture and expand these rare cells in vitro. This highlights the need of more studies to produce CTC/CDX models for these cancers. Additionally, the variety of techniques used to isolate and culture CTCs are both a challenge and an opportunity. Each method has its strengths and limitations, and their effectiveness can vary depending on the cancer type or/and the patient profile. This complexity poses challenges, but it also promotes the adaptation of approaches to meet specific needs. Exploring this diversity and working to harmonize or combine the best practices hold great potential. In parallel, researchers are testing organ-on-a-chip microfluidic systems to recreate aspects of the tumor microenvironment in vitro in order to provide the signals needed for CTC survival and proliferation in culture. Moreover, gentle, label-free isolation approaches and enriched culture conditions, such as low-oxygen environments, growth factor-rich media and 3D supports, also seem to improve CTC expansion, suggesting that refining these methodologies will increase the success of CTC/CDX model generation across a broader range of cancer types.

More studies are needed to deepen our understanding of the biological mechanisms underpinning these models. For instance, the role of oxidative stress in CTC survival remains poorly understood. Oxidative stress, which results from an imbalance between the production of free radicals and the body antioxidant defenses, could play a key role CTC adaptation and survival in their environment. More research is also needed on dormant CTCs. These cells enter a state of dormancy for varying lengths of time and are often responsible for cancer recurrence and late-stage metastases. Understanding why the current therapies do not kill these dormant cells and developing new, specific strategies to target them are major challenges. To address these questions, researchers are now exploiting single-cell multi-omics (integrating genomic, transcriptomic and epigenetic analyses of individual cells) to identify the molecular traits that enable some CTCs to survive treatment, enter dormancy (dormant DTCs) and eventually reawaken to seed metastases. Moreover, comparing the genetic profiles of CTCs, metastatic lesions and primary tumors from the same patient has revealed the emergence of new resistance-driving mutations in CTCs and metastases and their absence in the original tumor. These critical adaptations would have been missed by examining only the primary tumor.

Another major area is the study of CTC metastatic signatures to understand why some cancer types preferentially colonize specific organs, such as bones, liver, or lungs. Identifying the molecular and biological factors that guide the migration of cancer cells could help to predict and better treat metastases. Similarly, describing CTC epigenetic profiles, including DNA methylation and histone modifications, is another promising research avenue. These epigenetic changes play a key role in CTC plasticity and resistance, and studying them in depth could pave the way to new therapeutic approaches. In addition, new analytic techniques, such as integrated immunofluorescence with FISH (iFISH), enable the simultaneous visualization of CTC phenotypes and chromosomal abnormalities. This combined approach has revealed remarkable heterogeneity within the CTC population, distinguishing single CTCs from clustered CTC groups, and identifying epithelial *versus* mesenchymal traits. It also contributes to identify the subpopulations with the highest metastatic potential [119–121].

Moreover, the impact of mechanical forces, such as shear stress in the bloodstream, on CTC survival needs further investigation. These mechanical constraints, inherent to CTC circulation in the blood, influence their ability to survive in the blood, evade the immune system and colonize new sites. Exploring these dynamics could provide fresh insights to understand the biology of the metastatic cascade.

An exciting perspective is to use CTC and CDX models as parallel drug testing platforms alongside clinical trials, as part of a dynamic personalized medicine strategy. The idea of these co-clinical trials is to make the patient-derived model evolve in parallel with the patient’s disease, in order to test treatment combinations or alternatives and help to guide therapeutic decision-making in real time. Solid-tumor PDX models have already demonstrated the value of this approach, although their establishment can be time-consuming. Conversely, CDX models can be generated from blood samples obtained during the routine clinical follow-up, allowing researchers to capture the most recent molecular state of the disease as it adapts under therapeutic pressure.

Recent studies suggest that CTCs and metastatic lesions are more similar (genetically and phenotypically) to each other than to the primary tumor. This means that testing drugs using CTC-derived cell lines may predict the treatment response in patients with metastatic disease more accurately than models derived from the primary tumor. Besides drug sensitivity testing, CTC- and CDX-based systems can be used to track clonal evolution, emergence of resistance mechanisms, metabolic rewiring, and phenotypic transitions as they occur in the patient.

Importantly, these models are not only experimental tools, but could become true clinical supports. By providing a timely, patient-specific platform to evaluate therapeutic options, they could help clinicians to select the most effective treatment based on the evolving disease characteristics in each patient, rather than relying on biopsies obtained months or years earlier. Ultimately, integrating CTC and CDX models into clinical decision-making workflows may help to anticipate resistance, personalize therapeutic sequences, and improve outcomes for patients with metastatic cancer.

## Data Availability

No datasets were generated or analysed during the current study.

## Abbreviations

ALDH: Aldehyde dehydrogenase
bFGF: Basic fibroblast growth factor
CAM: Chorioallantoic membrane
CDK: Cyclin-dependent kinase
CDX: Circulating tumor cell-derived xenograft
cCSC: Circulating cancer stem cell
CO₂: Carbon dioxide
CTC: Circulating tumor cell
C-ETAC: Circulating ensembles of tumor-associated cells
DLA: Diagnostic leukapheresis
DMEM: Dulbecco’s modified eagle medium
DTC: Disseminated tumor cells
EGF: Epidermal growth factor
EGFR: Epidermal growth factor receptor
EMT: Epithelial-to-mesenchymal transition
EpCAM: Epithelial cell adhesion molecule
FACS: Fluorescence-activated cell sorting
HCC: Hepatocellular carcinoma
HNC: Head and neck cancer
MELK: Maternal embryonic leucine zipper kinase
MIC: Metastasis-initiating cell
NSCLC: Non-small cell lung cancer
O₂: Oxygen
PBMC: Peripheral blood mononuclear cell
PDAC: Pancreatic ductal adenocarcinoma
PDX: Patient-derived xenograft
RBC: Red blood cell
RPMI: Roswell park memorial institute (medium)
SCLC: Small cell lung cancer
TNBC: Triple-negative breast cancer
WNT: Wingless-related integration site (signaling pathway)
